# Cross-modal fusion of OCT and OCT angiography enface for improved diagnostics of diabetic retinopathy

**DOI:** 10.3389/fmed.2026.1900570

**Published:** 2026-09-29

**Authors:** Rashadul Hasan Badhon, Atalie Carina Thompson, Jennifer I. Lim, Theodore Leng, Minhaj Nur Alam

**Affiliations:** 1 Department of Electrical and Computer Engineering, University of North Carolina at Charlotte, Charlotte, NC, United States; 2 Department of Surgical Ophthalmology, Atrium-Health Wake Forest Baptist, Winston-Salem, NC, United States; 3 Department of Ophthalmology and Visual Science, University of Illinois at Chicago, Chicago, IL, United States; 4 Department of Ophthalmology, Stanford University School of Medicine, Stanford, CA, United States

**Keywords:** classification, crossmodal fusion, DR, OCT, OCTA, OCTA enface

## Abstract

**Introduction:**

Diabetic retinopathy (DR) is a leading cause of vision impairment worldwide, highlighting the need for accurate and accessible screening tools. Optical Coherence Tomography (OCT) provides high-resolution structural information of the retina, whereas OCT angiography (OCTA) offers complementary vascular information that is highly relevant for DR diagnosis.

**Methods:**

In this study, we propose a cross-modal fusion of OCT B-scans with single-channel enface OCTA using a bidirectional cross-modal attention network for automated DR classification. Two independent datasets, OCT500 and UIC, comprising 730 scans in total, were utilized to evaluate performance in within-dataset, combined-dataset, and cross-dataset generalization settings. A ConvNeXt V2 Large model trained solely on OCT images served as the unimodal baseline. In addition to ground-truth (GT) OCTA, we explored the use of translated (TR) OCTA generated from OCT scans, eliminating the requirement for dedicated OCTA hardware.

**Results:**

Experimental results demonstrated that cross-modal fusion consistently outperformed unimodal OCT classification across all evaluation scenarios. Fusion with GT OCTA improved classification accuracy and discriminative performance, while TR OCTA achieved comparable or superior results in most settings. Furthermore, TR OCTA improved sensitivity and cross-dataset generalization, indicating enhanced robustness to domain shifts.

**Discussion:**

These findings demonstrate that attention-based OCTOCTA enface fusion provides clinically meaningful improvements for DR detection and suggest that computationally generated OCTA can serve as a practical, low-cost alternative to hardware-acquired OCTA, enabling broader deployment of high-performance retinal screening systems in resource-limited clinical environments.

## 1 Introduction

Optical Coherence Tomography (OCT) is a non-invasive high-resolution imaging modality, using low-coherence interferometry to produce depth-resolved cross-sectional images of the microstructure of biological tissue with micrometer-scale resolution (1, 2). Since its initial demonstration, OCT has become popular in medical imaging due to its ability to visualize subsurface morphology in real time without contact or any invasive medical procedure. In the field of ophthalmology, OCT has revolutionized the assessment of retinal architecture and pathologies, enabling precise evaluation of retinal layers, photoreceptor integrity and subtle structural changes associated with major causes of vision loss such as age-related macular degeneration (AMD), diabetic retinopathy (DR) and glaucoma (3, 4).

Although traditional OCT captures structural retinal information, OCT Angiography (OCTA) extends OCT to visualize vascular networks non-invasively by detecting motion contrast between sequential B-scans at the same spatial location, thus highlighting blood flow dynamics without the need to introduce contrast agents (5). OCTA provides depth-resolved en face projections of retinal and choroidal microvasculature, offering new quantitative biomarkers such as vessel density, caliber etc which are critical for early detection and longitudinal monitoring of retinal vascular diseases (6, 7). The enface representation of OCTA data, projecting volumetric information onto a plane parallel to the retinal surface, is especially useful for visualizing top-down vascular maps and broader spatial patterns hidden within individual B-scans (8). OCTA enface, hence facilitates visualization of the vascular architecture in a global context, while individual OCT B-scans provide high-resolution structural contrast across depth.

Most of the commercially available devices produce OCT slices and OCTA enface. Not to mention that OCTA requires additional expensive hardware which is not always affordable or available at the local clinics. Hence, for retinal pathology detection, OCT has been widely utilized so far, despite the clear merits of OCTA (9–15). Given the additional vascular information present in OCTA, combining it with the already available OCT, disease prognosis and progression monitoring would be faster and more accurate, leading to better medical services. Thus cross-modal fusion comes to the scenario.

Recently, cross-modal retrieval has gained popularity due to the rapid increase in multimodal data thanks to the fast technological development in medical science. The idea of high level semantic homogeneity and low level expressive heterogeneity brought forth the necessity of combining multiple forms of data into one for better representation. Unimodal data handles information from one channel only, limiting any utilization from other modality data, thereby degrading potential performance improvement. Cross-modal image fusion has thus emerged as a pivotal paradigm in clinical diagnosis and research, as single-modality images often provide only fragmented representations of complex disease processes (16). This provides an opportunity to integrate complementary information from different imaging modalities to provide a more comprehensive understanding of the underlying pathology, leading to improved diagnostic accuracy and treatment planning (17, 18). Recent studies have demonstrated that deep learning-based multimodal fusion techniques have become powerful tools for medical image detection and prognosis.

In general, fusion approaches can be broadly categorized into three main schemes: early fusion (input level), intermediate fusion (feature-level) and late fusion (output level) (16, 19). Early fusion combines raw data from different modalities at the input level, allowing the network to learn joint representations from the outset. However, this approach may struggle to identify meaningful correlations between modalities when input characteristics differ substantially in terms of intensity, spatial patterns or noise profiles (20). Late fusion, on the other hand, combines predictions from modality-specific networks at the decision stage, offering flexibility but potentially sacrificing opportunities for deep feature interaction (21). Intermediate fusion, however, merges features at various depths within the network architecture and has demonstrated superior performance by enabling the model to learn hierarchical cross-modal correlations at different levels of abstraction (20, 22).

Recent advances have introduced attention mechanisms and Transformer architectures to address the limitations of traditional convolutional neural network (CNN) based fusion methods. CNNs excel at extracting local features through convolutional operations but have limited receptive fields that hinder their ability to capture long-range dependencies and global contextual information—essential for accurately integrating diverse modalities (23, 24). Vision transformers (ViTs) leverage multi-head self-attention mechanisms to model global context and long-distance relationships, proving highly effective in medical image fusion tasks (25, 26). Hybrid architectures combining CNNs and transformers have emerged as particularly promising, exploiting CNNs’ local feature extraction capabilities alongside transformers’ global modeling strengths (27, 28). Cross-attention mechanisms that are specifically designed for multimodal fusion have been proposed in recent studies to enhance complementary information extraction between modalities while suppressing redundant features (24). An ablation study was conducted to evaluate the contribution of the fusion strategy and cross-modal attention mechanism. Early fusion provided limited cross-dataset generalization, whereas the dual-stream cross-modal fusion architecture without attention showed a clear improvement, indicating that modality-specific feature extraction is beneficial for integrating OCT with OCTA features. The full model with cross-modal attention further improved overall discriminative performance (Supplementary Tables S1, S2).

In the context of neuroimaging, multimodal fusion of structural MRI and functional MRI using cross-attention mechanisms has significantly improved diagnostic accuracy for brain disorders (29). Similarly, in oncology, multimodal deep learning fusion combining radiological images with histopathological data has enhanced tumor classification and grading performance (30). These successes underscore the potential of sophisticated fusion strategies to leverage complementary relationships among clinical modalities for improved disease characterization and prognostic modeling. In ophthalmology, the integration of structural OCT and vascular OCTA information represents a natural and clinically meaningful fusion paradigm. Structural OCT provides detailed morphological information about retinal layers and tissue architecture, while OCTA reveals functional blood flow patterns and microvascular perfusion. Several studies have demonstrated that combining these complementary modalities improves classification performance for retinal diseases. For instance, multimodal training processes incorporating both enface OCT and OCTA have been successfully employed for automated artery-vein classification, where enface OCT provides vessel intensity profiles similar to near-infrared fundus images and OCTA contributes blood flow strength and vessel geometry features (31, 32).

Some recently published works have systematically evaluated different fusion strategies for OCTA layer combinations in retinal pathology classification. Intermediate fusion architectures, which merge features from multiple OCTA layers (superficial capillary plexus, deep capillary plexus, and choriocapillaris) at intermediate network depths, have achieved superior performance compared to early or late fusion approaches. The intermediate fusion strategy enables the model to learn and exploit complementary information from different vascular layers at appropriate levels of feature abstraction, addressing the limitation that early fusion may not allow CNNs to discriminate layer-specific pathological patterns effectively (20). Hierarchical fusion approaches combining structural OCT and OCTA with additional imaging modalities have also demonstrated advantages over simpler fusion schemes in DR detection (33). A recent study (34) showed that OCT coupled with multiple layer-specific projections feeding into a simple CNN can result in a better performance compared to early or late fusion. There have been other studies further demonstrating the potential of OCT and OCTA-based deep learning for automated DR assessment. Li et al. (35) evaluated a multilevel information-fusion framework directly on the OCT500 dataset and reported an accuracy of 88.1% and an AUC of 0.92 for distinguishing DR from normal eyes, outperforming conventional CNN architectures evaluated under the same protocol. Zang et al. (36) developed a three-dimensional deep-learning framework using volumetric structural OCT and OCTA and achieved an accuracy of 91.52% and an AUC of 0.96 for referable DR classification, demonstrating the benefit of jointly exploiting structural and vascular information. Similarly, Ryu et al. (37) reported AUC values ranging from 0.919 to 0.976 for OCTA-based DR and referable-DR detection, with comparable performance maintained during external validation. More recently, Shah et al. (38) evaluated cross-modal transfer learning with ophthalmic foundation models on OCT500 for four-class classification of normal, DR, AMD and other retinal diseases; their model achieved an accuracy of 0.8133±0.0470, exceeding the corresponding ImageNet-pretrained model (0.7600±0.0502).

However, challenges remain in effectively fusing the OCT and OCTA modalities that are available from commercial devices, since in a clinical setting the OCTA enface is often the only data available in addition to the usual OCT slices. Most of the studies conducted on DR detection used the same dataset for training and inference and sometimes take mutliple layer projections that are not available from regular OCT devices. Data heterogeneity resulting from modality-specific noise, resolution variations, and inconsistent annotations can impede performance and generalizability hence we need more than a simple CNN to process and extract the multimodal information. The requirement for paired multimodal data during both training and inference stages limits the broader adoption of fusion methods, as large-scale paired datasets are difficult to acquire (39). Moreover, maintaining significant cross-modal correlations during fusion while ensuring interpretability is crucial for clinical implementation hence adoption remains an open challenge (30). In parallel with advances in hardware and fusion methodologies, deep learning and computational methods have been increasingly applied to OCT and OCTA imaging to enable automated classification, segmentation, and synthetic image generation (9, 10). Deep learning models have demonstrated the feasibility of synthesizing OCTA-like vascular maps from structural OCT scans by learning reflectance—flow correlations (11). Building upon these foundations, our work investigates cross-modal fusion of structural OCT and enface OCTA for improved DR classification. Our novelty is several folds: (1) single channel enface OCTA is used as the 2nd modality to mimic a real life clinical settings of scarce data availability, (2) a proper cross-modal attention framework adapted for feature extraction and mixing, (3) We used 2 different datasets in different training arrangements to validate the performance against a based model classifier and (4) Use of translated (TR) OCTA enface replacing the ground truth (GT) OCTA enface generated from the OCT slices. This is significant from the perspective of affordable healthcare where instead of additional hardware and expenditure, we can utilize the already available dataset and get better performance. We have generated the TR OCTA enface after translating OCTA slices from the corresponding OCT slices using a conditional diffusion model, trained on OCT500 only and later inferred on UIC (12). We have added details of this study in the Supplementary Material.

Across our different training configurations, we found that the fusion of OCT and OCTA enface significantly improves performance over an unimodal model in terms of accuracy, precision, recall, F1, AUC and specificity. We also included confidence interval to validate the fusion process and generated results. On top of that we introduced TR OCTA as an alternative to acquire the 2nd modality data and the performance improved in most cases compared to the GT OCTA data.

The primary contributions of this work are fourfold. First, we demonstrated that cross-modal attention-based fusion of OCT with a single-channel OCTA enface consistently and substantially outperforms a strong unimodal ConvNeXt V2 Large baseline for DR classification across multiple within-dataset and cross-dataset scenarios. Second, we utilized TR OCTA enface, translated entirely from OCT slices via a conditional diffusion model, removing the dependency on physical OCTA hardware. The TR OCTA images used in this study were generated using our previously published OCT-to-OCTA translation framework. Their quality was validated using image quality metrics (SSIM, FID, and PCQI) together with quantitative retinal vascular biomarkers, including blood vessel density (BVD), blood vessel caliber (BVC), blood vessel tortuosity (BVT), and vessel perimeter index (VPI), on both OCT500 and UIC dataset against the GT. This TR OCTA fusion variant matches or exceeds GT OCTA fusion performance in the majority of experimental configurations. They also achieved higher sensitivity in cross-dataset generalization, making high-quality multimodal classification accessible with only a standard OCT scanner. Third, we provide a rigorous multi-scenario evaluation framework: spanning single or combined dataset training and bidirectional cross-dataset generalization across two independent cohorts (OCT500 and UIC) with distinct acquisition protocols and patient demographics. This was accompanied by 95% bootstrap confidence intervals, giving a statistically grounded assessment of both performance and uncertainty for this study. Fourth, by showing that a computationally synthesized second modality can substitute for expensive OCTA hardware without sacrificing diagnostic performance, this work establishes a clinically meaningful pipeline for affordable, high-sensitivity DR screening in resource-limited settings where OCTA devices are unavailable, offering a practical bridge between well-resourced specialist clinics and underserved primary care environments.

## 2 Materials and methods

### 2.1 Dataset

In this study, two independent retinal imaging datasets were used: OCT500 (40) and UIC. OCT500 is a publicly available dataset comprising OCT B-scan volumes and corresponding enface OCTA images acquired for 3mm and 6mm field of views (FoV). Out of 500 patients only 251 normal and 64 DR patients, each patient having a single scan volume, were used for this study since UIC has only normal and DR labels. UIC is another dataset collected at the University of Illinois Chicago, containing paired OCT and OCTA acquisitions from 64 normal patient scans and 351 DR patient scan volumes. Together, the two datasets provide complementary sources of multi-modal retinal data that differ in acquisition device, imaging protocol and patient demographics, yielding a combined pool of 315 normal and 415 DR scan volumes (730 total). For each scan volume, unimodal input consists of only OCT slices. On the other hand, the multi-modal input consists of one enface OCTA image and approximately 80 OCT B-scan slices (Figure 1) centred around the foveal region, giving a rich cross-sectional view of the macular tissue alongside the corresponding vascular projection. We also compared performance for TR OCTA enface replacing GT OCTA enface as an alternative, where we can utilize OCTA translation from OCT to enhance multimodal fusion scope. Stratified splits were applied at the Patient-level to prevent data leakage across partitions.

**Figure 1:**
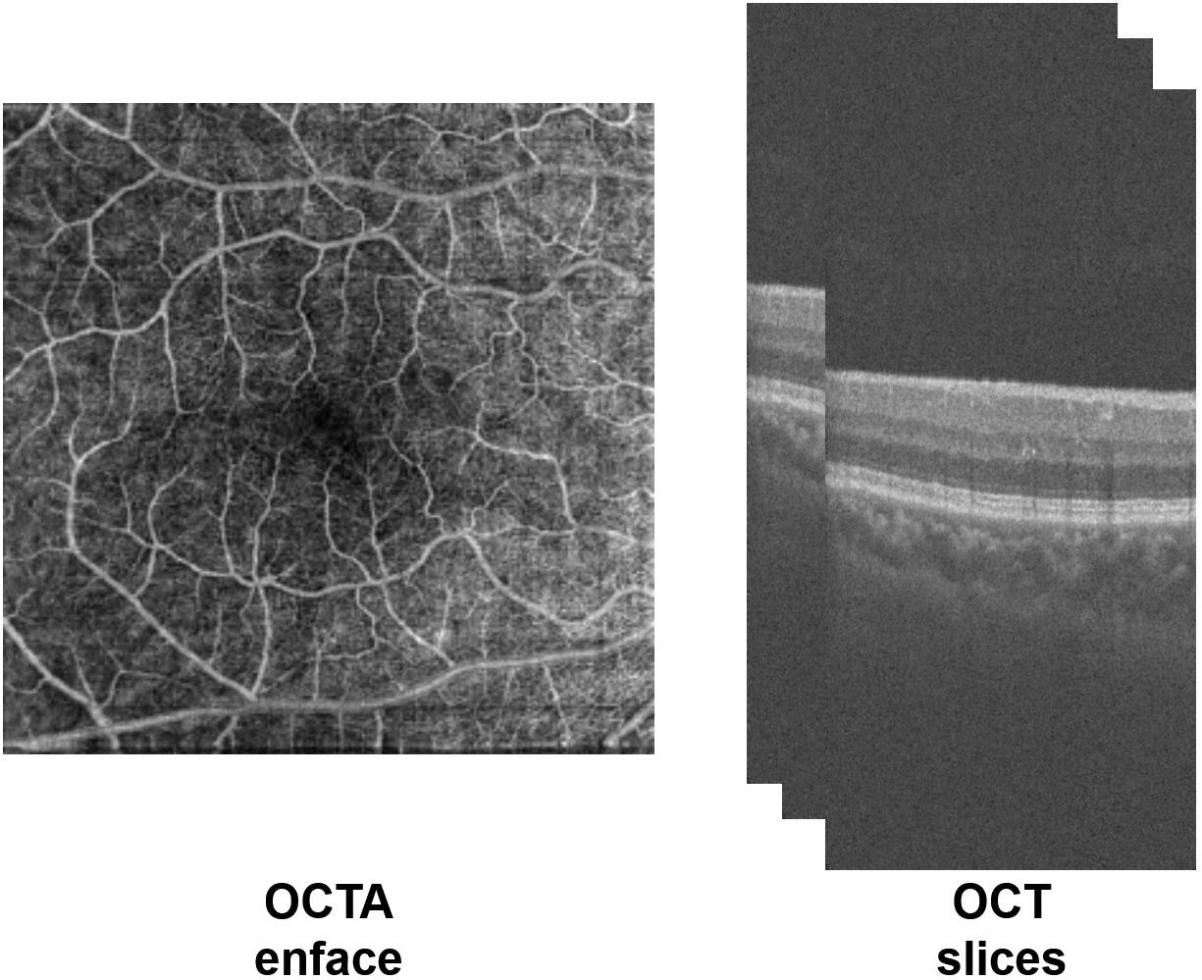
OCT and OCTA enface sample data

To systematically evaluate both the performance and the generalisation capability of the proposed models, five training and evaluation scenarios were planned. In the first two scenarios, each dataset was used independently: models were trained and tested exclusively on OCT500 or UIC data using an 80-10-10% train-validation-test split to establish dataset-specific performance benchmarks. In the third scenario, the two datasets were pooled into a single combined dataset, and the model was trained and tested on this merged collection, assessing whether a larger and more heterogeneous training set improves overall performance. The final two scenarios were designed to evaluate cross-dataset generalisation: in one, the model was trained on OCT500 (90-10% split) and tested on UIC; in the other, the training and test sets were reversed, with UIC used for training (90-10% split) and OCT500 for testing. These cross-dataset experiments probe how well models trained on one imaging environment transfer to an unseen acquisition setting, which is a critical consideration for clinical deployment. All experiments were conducted using two NVIDIA RTX A6000 GPUs, each with 48 GB of GPU memory. For the cross-dataset experiments, the dual-stream fusion model required approximately 2 min 21 s per training epoch on average, with a total training time of approximately 250 min for a complete training session. Inference on the complete test set required approximately 2–4 min to complete.

### 2.2 Model framework

To establish a single-modality baseline for comparison against the multi-modal fusion approach, we trained a ConvNeXt V2 Large network (41) independently on OCT B-scan images. The model was initialised with ImageNet-1k pretrained weights and its final classification head was replaced with a linear layer sized to the number of target classes. The network was trained end-to-end with a learning rate of $1\times 10^{-4}$. Input images were resized to 224 × 224 pixels and normalised, with no modality fusion or cross-modal attention applied. Performance was evaluated across the five scenarios mentioned above using accuracy, precision, recall, F1 score, AUC and specificity, while using the same data splits and evaluation protocol as CRD-Net, enabling direct comparison between single-modal and multi-modal strategies.

For the multimodal fusion model architecture, we adopted the Cross-modal Retinal Disease Diagnosis Network (CRD-Net) proposed by Liu et al. (42) as the fusion backbone to combine OCT B-scan and enface OCTA modalities. CRD-Net (Figure 2) is designed to explicitly model the spatial correspondence between paired multi-modal retinal images, rather than treating each modality independently.

**Figure 2:**
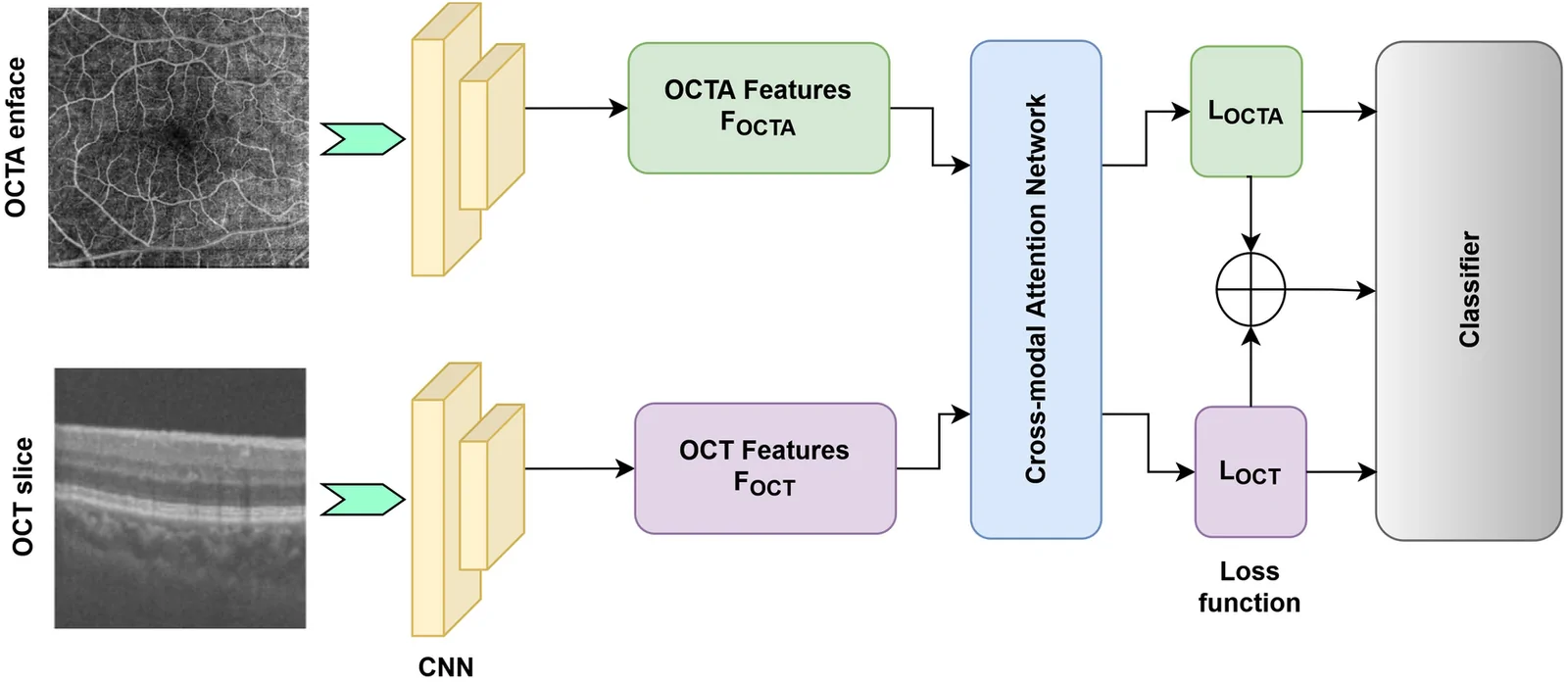
Cross-modal fusion framework.

The architecture consists of three principal components: (1) feature extraction via independent convolutional neural networks (CNNs), (2) a cross-modal Attention module that enables bidirectional feature exchange between modalities and (3) a multi-branch classifier trained with a composite loss function.

Each input modality: the OCT B-scan and the en face OCTA image, is processed by a separate CNN backbone to extract modality-specific instance-level feature representations. From the two modalities, two feature maps are extracted. The two backbones share the same architecture but maintain independent weights, allowing each stream to learn representations tailored to its modality’s characteristics. For all the experiments, we used ImageNet-pretrained weights as initialization. The final feature representations are taken from the last layer of each backbone, providing globally pooled, semantically rich feature maps prior to the fusion.

The attention module is motivated by the clinical workflow in which ophthalmologists identify a lesion in one imaging modality and then query the corresponding anatomical location in the other modality to corroborate their findings. This module consists of two sequential sub-components: a specific modal attention module and a cross-modal fusion module.

Within each stream, a multi-head self-attention mechanism is first applied to the feature maps to direct the network’s attention toward disease-relevant spatial regions within that modality. Specifically, each feature map is first projected via a 1 × 1 convolution, passed through the attention network to produce an intermediate feature and then further refined by a feed forward network with a residual connection back to the original feature map. This yields modality-specific attended outputs.

After per-modality attention, the fusion module performs bidirectional cross-modal querying. In the OCT branch, the attended OCT features are projected to form the key and value matrices, while the attended OCTA features serve as the query. A scaled dot-product attention is computed between these projections, and the result is added back via a residual connection to produce the OCT branch output. This allows the OCT branch to identify which of its own regions are most relevant to the lesion patterns observed in the enface OCTA. The symmetric operation is applied in the OCTA branch in a similar way to yield the OCTA branch output. Residual connections are used throughout to support stable gradient flow. The attention module is inserted at the output of the final layer as it ensures that the input features are sufficiently abstract and semantically rich before cross-modal interaction is performed. Following the attention module, the cross-attended feature maps are concatenated to form a unified multi-modal representation, which is passed to a fully connected classification head. Two additional single-modality classifiers are simultaneously trained on each stream’s attended features independently. The total training objective is the sum of three cross-entropy losses: one from the fused multi-modal branch, one from the OCT-only branch, and one from the OCTA-only branch, all jointly optimised via Stochastic Gradient Descent (SGD). The models were trained using standard cross-entropy loss first and later using weighted cross-entropy loss. Stratified patient-level splitting was used to preserve the class distribution while preventing leakage between training, validation, and test partitions. This composite loss ensures balanced supervision across both modalities, discouraging over-reliance on either single stream and reducing modality-specific bias during training.

All models were implemented in PyTorch. Following the original CRD-Net configuration, the optimiser was SGD with momentum 0.9 and weight decay $1\times 10^{-4}$. The initial learning rate was set to $1\times 10^{-4}$ and decayed by a factor of 0.1 at epochs 50 and 100, training for a total of 100 epochs with a batch size of 32. Input images from both modalities were augmented identically using random horizontal flipping (probability 0.5) and random rotation within ±30${}^{\circ }$. All backbone weights were initialised from ImageNet pre-trained checkpoints.

## 3 Results

We compared the classification performance of both the unimodal baseline and the proposed cross-modal fusion model across three within-dataset training configurations: OCT500, UIC and Combined and two cross-dataset generalization scenarios: OCT500-trained model tested on UIC, and UIC-trained model tested on OCT500. We reported performance using accuracy, precision, recall, F1-score, AUC and specificity. For the cross-modal fusion model, all metrics are accompanied by 95% bootstrap confidence intervals (CIs) computed over 1,000 resampling iterations to provide reliable estimates of statistical uncertainty.

### 3.1 Unimodal baseline performance

Table 1 presents the classification performance of the unimodal ConvNeXt V2 Large baseline model for different training arrangements. When trained and tested within the same dataset, the model achieves an accuracy of 0.8634 on OCT500 and 0.9621 on UIC. However, the OCT500 model shows a clear precision-recall imbalance, with high precision (0.9391) and specificity (0.9934) but low recall (0.3810), resulting in a modest F1-score of 0.5420. The UIC model, by contrast, is more balanced, with an F1-score of 0.9775. The combined dataset achieves an accuracy of 0.9221 and F1-score of 0.9281, suggesting that pooling data helps offset the recall degradation seen with OCT500 alone. Cross-dataset generalization reveals a significant performance drop: when trained on OCT500 and tested on UIC, accuracy falls to 0.5735; precision remains high (0.9927), but recall collapses to 0.4994, giving an F1-score of 0.6645. This suggests the OCT500-trained model rarely misclassifies healthy cases but misses a large share of true DR cases on UIC. The reverse arrangement, UIC trained and tested on OCT500, yields an accuracy of 0.7048 with recall recovering to 0.7284, but precision drops sharply to 0.3814, giving a low F1-score of 0.5007, indicating a tendency toward over-prediction of DR.

**Table 1:** Unimodal baseline (ConvNeXt V2 Large) classification performance.

Training arrangement	Accuracy	Precision	Recall	F1-score	AUC	Specificity
OCT500	0.8634 (0.8496–0.8766)	0.9391 (0.9067–0.9657)	0.3810 (0.3362–0.4211)	0.5420 (0.4950–0.5826)	0.7994 (0.7763–0.8225)	0.9934 (0.9896–0.9962)
UIC	0.9621 (0.9555–0.9681)	0.9731 (0.9670–0.9792)	0.9818 (0.9769–0.9865)	0.9775 (0.9734–0.9812)	0.9882 (0.9853–0.9911)	0.8607 (0.8299–0.8895)
Combined	0.9221 (0.9151–0.9284)	0.9751 (0.9693–0.9803)	0.8854 (0.8742–0.8955)	0.9281 (0.9212–0.9340)	0.9764 (0.9727–0.9801)	0.9703 (0.9636–0.9769)
OCT500 train UIC test	0.5735 (0.5682–0.5786)	0.9927 (0.9913–0.9940)	0.4994 (0.4936–0.5052)	0.6645 (0.6592–0.6698)	0.9003 (0.8969–0.9037)	0.9797 (0.9760–0.9834)
UIC train OCT500 test	0.7048 (0.6990–0.7103)	0.3814 (0.3720–0.3907)	0.7284 (0.7165–0.7407)	0.5007 (0.4912–0.5100)	0.7939 (0.7862–0.8016)	0.6988 (0.6921–0.7050)

Values in parentheses represent 95% bootstrap confidence intervals (1,000 iterations).

### 3.2 Cross-modal fusion performance with GT OCTA

Table 2 presents the performance of the cross-modal fusion model using GT OCTA enface images. Our results indicate that within-dataset performance is substantially improved over the unimodal baseline. On OCT500, the fusion model achieves an accuracy of 0.8788 and an AUC of 0.9719, with perfect precision and specificity (both 1.0000) but limited recall (0.4286), yielding an F1-score of 0.6000. On UIC, performance is near-ceiling, with an accuracy of 0.9971, an AUC of 1.0000, and near-perfect precision (0.9966) and recall (1.0000), giving an F1-score of 0.9983. The combined dataset yields an accuracy of 0.9730, with perfect precision (1.0000) and strong recall (0.9524), producing an F1-score of 0.9756.

**Table 2:** Cross-modal fusion classification performance (GT OCTA).

Training arrangement	Accuracy	Precision	Recall	F1-score	AUC	Specificity
OCT500	0.8788 (0.8646–0.8896)	1.0000 (1.0000–1.0000)	0.4286 (0.3857–0.4695)	0.6000 (0.5567–0.6390)	0.9719 (0.9656–0.9774)	1.0000 (1.0000–1.0000)
UIC	0.9971 (0.9954–0.9989)	0.9966 (0.9942–0.9986)	1.0000 (1.0000–1.0000)	0.9983 (0.9971–0.9993)	1.0000 (1.0000–1.0000)	0.9824 (0.9702–0.9928)
Combined	0.9730 (0.9688–0.9768)	1.0000 (1.0000–1.0000)	0.9524 (0.9450–0.9593)	0.9756 (0.9717–0.9792)	0.9944 (0.9932–0.9953)	1.0000 (1.0000–1.0000)
OCT500 train UIC test	0.8746 (0.8713–0.8783)	0.8709 (0.8671–0.8742)	1.0000 (1.0000–1.0000)	0.9310 (0.9288–0.9329)	0.9651 (0.9631–0.9671)	0.1869 (0.1767–0.1971)
UIC train OCT500 test	0.8345 (0.8297–0.8386)	0.8252 (0.8059–0.8436)	0.2350 (0.2238–0.2466)	0.3658 (0.3516–0.3805)	0.7216 (0.7137–0.7299)	0.9873 (0.9857–0.9888)

Values in parentheses represent 95% bootstrap confidence intervals (1,000 iterations).

In the cross-dataset generalization, the OCT500-trained model tested on UIC achieves an accuracy of 0.8746 and an AUC of 0.9651, with recall reaching 1.0000, but at the cost of specificity, which falls sharply, indicating the model over-predicts DR cases when applied to UIC. The resulting F1-score is 0.9310, inflated by the strong recall despite this over-prediction tendency. The UIC-trained, OCT500-tested case shows the opposite pattern: accuracy is 0.8345 with an AUC of 0.7216, but recall drops sharply to 0.2350 while specificity remains high (0.9873), giving a low F1-score of 0.3658. This indicates the UIC-trained model becomes overly conservative on OCT500, missing most true DR cases.

### 3.3 Cross-modal fusion performance with TR OCTA

Table 3 presents the cross-modal fusion performance when TR OCTA enface is utilized instead of GT OCTA. Within-dataset performance is markedly higher than both the unimodal baseline and the GT OCTA fusion variant. On OCT500, the model achieves an accuracy of 0.9697 and an AUC of 0.9897, with perfect precision and specificity but a slightly lower recall of 0.8571, giving a strong F1-score of 0.9231. On UIC, performance is similarly high, with perfect recall (1.0000) and an F1-score of 0.9823, though specificity is comparatively lower at 0.8148. The combined dataset achieves the strongest overall metrics: accuracy 0.9820, near-perfect recall (0.9997), and AUC 0.9991, demonstrating that TR OCTA provides more consistent and informative cross-modal features for classification. Cross-dataset generalization is also improved under TR OCTA compared to the base model. The OCT500-trained model tested on UIC achieves an accuracy of 0.8829 and a recall of 0.8747, with high precision (0.9852) and specificity (0.9279), yielding a well-balanced F1-score of 0.9266 and indicating substantially better sensitivity than the GT OCTA counterpart. The UIC-trained model tested on OCT500 remains the most challenging scenario, yielding an accuracy of 0.7005 and an AUC of 0.8047; recall recovers to 0.7872, but precision drops to 0.3843, giving an F1-score of 0.5165. Even so, the TR OCTA fusion model still outperforms the unimodal baseline in this direction.

**Table 3:** Cross-modal fusion classification performance (TR OCTA).

Training arrangement	Accuracy	Precision	Recall	F1-score	AUC	Specificity
OCT500	0.9697 (0.9630–0.9761)	1.0000 (1.0000–1.0000)	0.8571 (0.8286–0.8853)	0.9231 (0.9062–0.9391)	0.9897 (0.9862–0.9931)	1.0000 (1.0000–1.0000)
UIC	0.9699 (0.9638–0.9753)	0.9652 (0.9589–0.9720)	1.0000 (1.0000–1.0000)	0.9823 (0.9790–0.9858)	1.0000 (1.0000–1.0000)	0.8148 (0.7832–0.8492)
Combined	0.9820 (0.9786–0.9853)	0.9695 (0.9634–0.9750)	0.9997 (0.9995–0.9999)	0.9844 (0.9812–0.9872)	0.9991 (0.9988–0.9994)	0.9587 (0.9506–0.9659)
OCT500 train UIC test	0.8829 (0.8795–0.8862)	0.9852 (0.9837–0.9867)	0.8747 (0.8705–0.8786)	0.9266 (0.9241–0.9290)	0.9595 (0.9573–0.9615)	0.9279 (0.9209–0.9347)
UIC train OCT500 test	0.7005 (0.6950–0.7057)	0.3843 (0.3746–0.3930)	0.7872 (0.7752–0.7984)	0.5165 (0.5064–0.5256)	0.8047 (0.7989–0.8110)	0.6784 (0.6716–0.6841)

Values in parentheses represent 95% bootstrap confidence intervals (1,000 iterations).

Supplementary Figures S2–S4 depict the confusion matrices and Supplementary Figures S5–S7 represent ROC curves for all the training scenarios where Normal is set as label “0” and DR is set as label “1”. Moreover, Supplementary Figures S1, S4 show base model performance; Supplementary Figures S2, S5 show cross-modal performance fusion pertaining to GT OCTA and Supplementary Figures S3, S6 show cross-modal fusion performance for TR OCTA.

ROC curves of the unimodal baseline model for within-dataset evaluations yield moderate to strong discriminative performance. The UIC and Combined curves rise sharply toward the upper-left corner at low false positive rates, indicating reliable separation of DR from NORMAL cases when training and test distributions are matched. In contrast, the OCT500 curve exhibits a more gradual ascent with visible irregularities. Under cross-dataset conditions, the OCT500-trained model tested on UIC achieves an AUC of 0.9003 despite a notable drop in recall. The UIC-trained model tested on OCT500 yields the lowest AUC of 0.7939, with a curve that departs more slowly from the diagonal, consistent with the greater domain gap when transferring from UIC to OCT500. On the other hand, ROC curves of the cross-modal fusion model with GT OCTA within-dataset performance is substantially stronger than the unimodal baseline. The UIC curve reaches the upper-left corner almost immediately, indicating near-perfect class separation, while the OCT500 and Combined curves both exhibit steep initial ascents before plateauing near maximum sensitivity. In the cross-dataset setting, the OCT500-trained model tested on UIC has the curve climbing sharply at low false positive rates before gradually leveling off. The UIC-trained model tested on OCT500 yields the lowest AUC of 0.7216, with a noticeably shallower curve that tracks closer to the diagonal. Lastly, for TR OCTA enface, across all within-dataset configurations, the curves rise steeply and hug the upper-left boundary, a consistent improvement over the GT OCTA variant. The Combined curve is nearly indistinguishable from a perfect classifier across the full range of thresholds. In the cross-dataset scenarios, the OCT500-trained model tested on UIC achieves an AUC of 0.9595, with a curve shape closely resembling its GT OCTA counterpart but with a more favorable balance between sensitivity and specificity, consistent with the sharp reduction in over-prediction observed in Table 3. The UIC-trained model tested on OCT500 achieves an AUC of 0.8047, representing a meaningful improvement over the GT OCTA cross-dataset result of 0.7216; while the curve still departs more gradually from the diagonal than in the within-dataset cases, the overall shape indicates that TR OCTA features generalize more effectively across acquisition domains than GT OCTA features.

Due to the nature of the unbalanced data distribution, we ran an ablation study with weighted cross-entropy loss for both base model and fusion model (GT OCTA enface) for cross-dataset scenarios (Tables 4, 5). When trained on OCT500 and tested on UIC, cross-modal fusion substantially improved cross-dataset performance, increasing accuracy from 0.6675 to 0.9454, recall from 0.6084 to 0.9632, F1-score from 0.7558 to 0.9676, and AUC from 0.9290 to 0.9832. For the reverse UIC-to-OCT500 evaluation, the fusion model similarly increased accuracy and AUC, indicating improved overall discrimination. However, the model exhibited a highly conservative operating point for the DR-positive class, with precision and specificity reaching 1.0000 while recall decreased to 0.0721. Consequently, the F1-score also decreased to 0.1346.

**Table 4:** Unimodal baseline (ConvNeXt V2 Large) classification performance with weighted loss.

Training arrangement	Accuracy	Precision	Recall	F1-score	AUC	Specificity
OCT500 train UIC test	0.6675 (0.6623–0.6729)	0.9975 (0.9967–0.9982)	0.6084 (0.6025–0.6143)	0.7558 (0.7512–0.7603)	0.9290 (0.9261–0.9319)	0.9915 (0.9889–0.9938)
UIC train OCT500 test	0.5469 (0.5407–0.5529)	0.2914 (0.2841–0.2989)	0.8596 (0.8501–0.8697)	0.4353 (0.4266–0.4440)	0.7664 (0.7596–0.7736)	0.4671 (0.4603–0.4739)

Values in parentheses represent 95% bootstrap confidence intervals (1,000 iterations).

**Table 5:** Cross-modal fusion classification performance (GT OCTA) with weighted loss.

Training arrangement	Accuracy	Precision	Recall	F1-score	AUC	Specificity
OCT500 train UIC test	0.9454 (0.9427–0.9479)	0.9720 (0.9700–0.9739)	0.9632 (0.9610–0.9655)	0.9676 (0.9659–0.9690)	0.9832 (0.9821–0.9843)	0.8480 (0.8381–0.8580)
UIC train OCT500 test	0.8115 (0.8067–0.8161)	1.0000 (1.0000–1.0000)	0.0721 (0.0655–0.0798)	0.1346 (0.1230–0.1478)	0.8556 (0.8505–0.8611)	1.0000 (1.0000–1.0000)

Values in parentheses represent 95% bootstrap confidence intervals (1,000 iterations).

## 4 Discussion

In this study, we combined OCT B-scans and single-channel OCTA enface images through a bidirectional cross-modal attention fusion network to classify DR against normal cases. The OCT and OCTA enface data used in this study were acquired as paired volumes (OCT500 only, UIC has enface instead of paired OCTA slices) using the Optovue platform. In this system, OCTA flow information is derived from repeated OCT B-scans acquired at the same retinal locations, and the orthogonally acquired volumetric scans are automatically registered and merged using the manufacturer’s Motion Correction Technology. Therefore, the structural OCT and OCTA data share a common volumetric coordinate system and field of view. We consequently did not perform an additional inter-modality registration step before model training. Residual misalignment may nevertheless arise from ocular motion, imperfect motion correction, segmentation errors or projection artifacts. Such inconsistencies could reduce the correspondence between OCT and OCTA enface features and cause the cross-modal attention module to assign importance to spatially inconsistent features, thereby reducing the effectiveness of multimodal fusion. There’s a large scope for data coherency improvement to make this study more clinically adaptable.

We evaluated performance across five experimental configurations spanning within-dataset, combined dataset and cross-dataset scenarios. Furthermore, we introduced a TR OCTA variant configuration that removes the cost, time consumption and hardware dependency for OCTA data collection. Taken together, the results confirm that cross-modal fusion consistently and substantially outperforms unimodal OCT classification and that the TR OCTA is another potential alternative for improved performance.

From Tables 1–3, the benefit of combining structural and vascular retinal information is particularly evident in discriminative performance and sensitivity. OCT B-scans provide depth-resolved structural information regarding retinal architecture and pathological changes, whereas OCTA enface images provide complementary information regarding retinal microvascular topology, including vessel-density alterations, capillary dropout, and abnormalities of the foveal avascular region. Combining these modalities therefore allows the classifier to form a richer disease representation than that available from either structural or vascular information alone. This effect is particularly apparent with TR OCTA fusion, for which recall exceeded that of the OCT-only baseline in all five evaluation configurations. This is clinically relevant for a screening-oriented system, where missed DR cases represented by false-negative classifications are an important consideration.

The unimodal base model (Table 1), trained solely on OCT B-scans, establishes the performance achievable from structural imaging alone under our experimental conditions. On OCT500, it achieves an accuracy of 0.8634 and an F1-score of 0.5420. Although precision and specificity are high, recall is only 0.3810, indicating that a substantial proportion of DR-positive instances are missed at the selected operating threshold. In contrast, the UIC model shows considerably stronger within-dataset performance, with an accuracy of 0.9621, recall of 0.9818, and F1-score of 0.9775. The combined dataset results suggest that exposure to a larger and more heterogeneous training population can reduce the pronounced precision-recall imbalance observed on OCT500 alone. These differences are also influenced by the markedly different class distributions of the two cohorts. OCT500 contains substantially more Normal than DR subjects, whereas UIC is strongly DR dominant, creating different learning conditions for the minority and majority classes.

When GT OCTA is introduced as the second modality, the classification characteristics change substantially (Table 2). On OCT500, accuracy increases while AUC increases markedly from 0.7994 to 0.9719. The comparatively modest change in fixed-threshold accuracy together with the large improvement in AUC indicates that cross-modal fusion produces substantially stronger separation between DR and Normal samples across classification thresholds, even though the operating point remains affected by the imbalanced class distribution. On UIC, GT fusion achieves an accuracy of 0.9971 and AUC of 1.0000, indicating nearly complete separation of the two classes under the matched acquisition and training conditions. The combined dataset similarly achieves an accuracy of 0.9730, an F1-score of 0.9756, and an AUC of 0.9944. These findings support the utility of combining OCT and OCTA enface, while also demonstrating that improvements in threshold-independent discrimination do not necessarily translate uniformly to every threshold-dependent metric. Our OCT500 GT fusion AUC of 0.9719 also compares favorably with previously reported OCT/OCTA-based DR classification studies discussed above, although differences in patient populations, disease definitions, imaging inputs, and evaluation protocols preclude direct head-to-head comparison.

Lastly, we replaced GT OCTA with TR OCTA generated from the corresponding OCT data using the diffusion-based translation framework. The resulting model maintained strong classification performance across the majority of configurations while exhibiting several important differences from GT OCTA fusion (Table 3). On OCT500, accuracy, recall, F1-score and AUC increase compared to the GT OCTA configuration. On UIC, both GT and TR fusion achieve a recall and AUC of 1.0000; however, GT fusion retains higher accuracy and specificity, indicating that TR OCTA utilized fusion does not universally outperform the GT OCTA configuration. In the combined training arrangement, TR OCTA achieves an accuracy of 0.9820, recall of 0.9997, F1-score of 0.9844, and AUC of 0.9991, while GT OCTA retains higher precision and specificity. Thus, TR OCTA provides competitive or improved classification performance in several configurations, but its benefit is metric- and dataset-dependent rather than uniform.

One possible explanation for the strong TR OCTA classification performance is that the translation model provides a learned vascular representation derived directly from the OCT input. Because the translation network was trained to model structural-to-vascular correspondences across paired OCT/OCTA samples, its output may suppress some acquisition-specific variability while retaining vascular patterns that are useful for classification. This may in turn provide the fusion network with a more regularized representation than individual GT OCTA acquisitions in some settings. However, this mechanism was not directly evaluated in the present study and should therefore be interpreted as a possible explanation rather than an established property of TR OCTA. Importantly, the translation model was trained exclusively using OCT500 and was not trained on UIC, making its performance on UIC relevant to the generalization capability of the translation pipeline and partially the reason being the UIC dataset not having the paired GT OCTA slices like OCT500.

We understand that within-dataset performance is necessary, however not sufficient, for clinical deployment. So, we also designed cross dataset experiments since they are the most diagnostically relevant results in this study. When the OCT500-trained model is evaluated on UIC, the unimodal baseline reaches an accuracy of only 0.5735 despite an AUC of 0.9003. Precision remains high at 0.9927, whereas recall falls to 0.4994, resulting in an F1-score of 0.6645. The relatively high AUC together with poor fixed-threshold classification indicates that the model retains substantial ranking ability across thresholds but operates poorly at the selected decision threshold under the shifted UIC distribution. GT OCTA fusion substantially increases accuracy to 0.8746 and AUC to 0.9651, and recall reaches 1.0000. However, specificity decreases markedly to 0.1869, indicating extensive over-prediction of DR among Normal UIC samples. This pattern is directly visible in the corresponding confusion matrix, where no DR samples are missed but a large number of Normal samples are classified as DR. Thus, the GT OCTA model improves sensitivity and overall discrimination in this direction, but its fixed-threshold operating point remains poorly calibrated to the target distribution.

The TR OCTA model provides a substantially more balanced operating point in the same OCT500→UIC evaluation. Although its recall is lower than the perfect recall obtained with GT OCTA, specificity increases dramatically while maintaining high sensitivity, indicating a considerably better balance between detection of DR and rejection of Normal cases while AUC remains close to that of GT fusion. The reverse UIC→OCT500 direction remains more challenging. GT fusion achieves relatively high accuracy and specificity but low recall, indicating a conservative DR-positive operating point. TR fusion increases recall, F1-score and AUC although accuracy and specificity falls. Compared with the OCT-only baseline, the TR model therefore provides improved sensitivity, F1-score, and a modest improvement in AUC, rather than a uniform improvement across all metrics. These opposing cross-dataset error patterns suggest that both domain shift and differences in class prevalence affect the fixed decision threshold and should be considered during future model calibration.

To assess whether the observed AUC improvement in the OCT500-trained model evaluated on UIC was statistically significant, we additionally performed DeLong’s test for the ROC curves generated from the same held-out evaluation samples. The unimodal baseline achieved an AUC of 0.9003, whereas the GT OCTA fusion model achieved an AUC of 0.9651. DeLong’s test indicated that the improvement was statistically significant (p<0.05), supporting the conclusion that the increase in discriminative performance associated with cross-modal fusion was unlikely to be attributable to random variation alone. This statistical comparison complements the 95% bootstrap confidence intervals reported for the classification metrics.

Since the datasets are inherently imbalanced, we additionally evaluated weighted cross-entropy loss for the two cross-dataset experiments (Tables 4, 5). For OCT500→UIC, applying GT OCTA cross-modal fusion under the same weighted-loss condition increases accuracy,recall, F1-score and AUC, while maintaining a specificity of 0.8480. This represents one of the strongest demonstrations of the benefit of multimodal fusion under cross-dataset evaluation. In contrast, weighted fusion in the UIC→OCT500 direction increases accuracy and AUC but shifts the classifier toward a highly conservative DR-positive operating point: precision and specificity reach 1.0000 while recall and F1-score decrease. Therefore, weighted loss can improve class discrimination but does not by itself guarantee an appropriate operating threshold after domain transfer. Threshold calibration or target-domain calibration strategies may consequently be necessary in addition to class weighting.

The ablation experiments further clarify the contribution of the fusion architecture and cross-modal attention. Under the weighted OCT500→UIC setting, early fusion achieves an accuracy of 0.7040, F1-score of 0.8047, and AUC of 0.7377 (Supplementary Table S1). Separating the modalities into independent streams and performing fusion without attention increases these values (Supplementary Table S2). The complete cross-modal attention model further improves accuracy, F1-score and AUC (Table 5). These progressive gains support the use of modality-specific feature extraction followed by attention-mediated feature interaction rather than simple input-level fusion. In the reverse UIC→OCT500 direction, AUC similarly increases with early fusion to without attention and with the full attention model. However, recall at the fixed threshold decreases, again demonstrating that improvements in ranking performance can coexist with poor target-domain threshold calibration.

We additionally performed an exploratory domain-adaptation experiment in the OCT500→UIC configuration using singular value decomposition to replace lower-level target-domain information (Supplementary Table S3). This approach did not improve the full weighted-fusion result. After adaptation, accuracy was 0.8500, AUC 0.8231, and specificity only 0.2201, compared with 0.9454, 0.9832, and 0.8480, respectively, for the corresponding full weighted fusion model without this adaptation procedure. Recall remained high at 0.9649, suggesting that the adapted representation strongly favored identification of DR but failed to retain adequate separation of Normal UIC samples. One plausible explanation is that the SVD-based replacement reduces global inter-dataset differences without preserving class-conditional information required to distinguish Normal from DR within the target dataset. These results should not be interpreted as evidence against domain adaptation in general; rather, they indicate that simple lower-level feature replacement is insufficient for the present domain shift. More principled strategies such as domain-adversarial feature learning, class-conditional alignment, or domain-generalization approaches should therefore be evaluated in future work.

Although the proposed fusion framework substantially improved cross-dataset performance compared with the unimodal baseline, a noticeable performance gap remained relative to within-dataset evaluation. Several factors likely contributed to this reduction in generalization. First, the OCT500 and UIC datasets were acquired using different OCT/OCTA systems with distinct imaging protocols, scan quality, preprocessing pipelines, and noise characteristics, resulting in a significant domain shift. Second, the disease distributions differ considerably between the two cohorts. OCT500 contains relatively few DR cases and a larger proportion of normal subjects, whereas the UIC dataset predominantly consists of diabetic retinopathy patients with varying disease severities and varying image quality. Consequently, models trained on one dataset encounter substantially different feature distributions when evaluated on the other. Third, differences in patient demographics, image quality, motion artifacts, and annotation practices may further increase inter-dataset variability. Finally, the translated OCTA model was trained exclusively on OCT500 data, which may also introduce a distribution mismatch when generating TR OCTA for UIC images. Collectively, these factors limit cross-dataset generalization despite the improvements provided by multimodal fusion. Future work will focus on incorporating larger multi-center datasets, domain adaptation strategies, domain generalization techniques, and scanner-independent normalization methods to improve robustness across diverse clinical environments.

The reduced performance observed in the cross-dataset evaluation highlights the effect of reduced model generalizability. A potential solution to this problem can be domain-adaptation strategies, including domain-adversarial feature learning and feature-distribution alignment, to encourage representations that remain discriminative for DR while being less dependent on the source dataset. However, we got an interesting result from our ablation study on the OCT500-to-UIC experimental case (Supplementary Table S3) where accuracy dropped after using singular value decomposition (SVD) to replace lower-level information of UIC with OCT500. The plausible explanation can be that the many Normal samples are also being detected as DR (low specificity) and the domain shift is domain-level rather than conditional. It can reduce domain differences but probably does not explicitly preserve the distinction between UIC Normal and UIC DR. Hence domain adaptation can only help when cross datasets have enough common retinal pathologies to detect the anomaly rather than detecting domain level information. Still with further research such approaches may be particularly valuable for resource-limited settings, where imaging devices and patient populations may differ considerably from those represented in the original training data.

Although we got good results for both GT OCTA and TR OCTA as expected, several limitations constrain the generalizability of the current findings. The dataset sizes remain modest: only 64 DR and 251 Normal patients in OCT500, and 415 scans in UIC limiting the diversity of disease presentations seen during training. Most publicly available OCT datasets are limited in size and phenotypic diversity, which create a bottleneck for generalization. The current study addresses only binary DR-vs.-NORMAL classification; extending to DR severity grading, which is the clinically actionable task for treatment decisions, would require substantially more labelled data at each severity level. Moreover, the translation algorithm has more scope for improvement in the TR OCTA performance in this sector. Although TR-OCTA preserves the major vascular architecture and disease-related vascular patterns, it does not perfectly reproduce all features observed in GT OCTA. Small capillary branches, regions with low flow signals, and subtle local vascular density variations may not be fully recovered by the translation model. Our previous quantitative validation demonstrated that vessel density exhibited the largest deviation between TR OCTA and GT OCTA, whereas vessel tortuosity and vessel perimeter index remained considerably more consistent across datasets. These findings suggest that TR OCTA is better at preserving global vascular morphology than very fine microvascular details. An important limitation of TR OCTA is the potential for generative hallucination. Because OCTA contains flow-related vascular information that is not directly observed in OCT, OCT-to-OCTA translation is inherently dependent on the learned training data. Consequently, the generative model may produce convincing vascular structures that are not present in the patient’s true anatomy, suppress existing pathological vessels or alter clinically meaningful characteristics. Such errors are particularly concerning because generated structures may appear visually realistic and may not be adequately identified by conventional image-quality metrics. Previous studies of generative medical-image translation and diffusion-based reconstruction have similarly demonstrated that anatomically incorrect or hallucinated structures can occur despite realistic image appearance. Although our previously developed diffusion-based OCT-to-OCTA framework showed lower hallucination scores than the GAN-based approach, hallucinations were not completely eliminated. Therefore, TR OCTA in the present study should be regarded as an auxiliary representation for disease-classification research rather than a substitute for acquired OCTA or a definitive representation of patient-specific vasculature. Before clinical translation, future studies should perform masked expert assessment, vessel- and lesion-level correspondence analysis with ground-truth OCTA, uncertainty estimation, evaluation across rare and out-of-distribution pathologies, and automated quality-control or hallucination-detection procedures to identify potentially unreliable translated images. Despite these limitations, the translated images retain sufficient clinically relevant vascular information to enhance multimodal classification performance. Different dataset having different scanners and acquisition protocol along with different population also affect the model performance specially the UIC dataset since its quality is inconsistent unlike OCT500.

The primary clinical significance of this work is not just the performance numbers, but the pipeline architecture they validate: a system that requires only a standard OCT scanner at the point of care, uses a learned translation to synthesize vascular information and applies attention-based fusion to produce a classification output that approaches the performance of a system with full OCTA hardware. Clinical fidelity of this work is supported by the patient-specific conditional translation process, paired OCT–OCTA training data, patient-level data separation, and cross-dataset evaluation. In addition, the fusion classifier retains the original OCT stream and does not rely exclusively on the synthesized OCTA image. Bidirectional cross-modal attention allows vascular features from TR OCTA to be evaluated in relation to structural features present in the corresponding OCT B-scans. Nevertheless, the current evaluation demonstrates diagnostic utility rather than definitive anatomical fidelity. Direct validation by retinal specialists, lesion-level correspondence analysis between GT and TR OCTA, and prospective evaluation across scanners and clinical populations will be required before translated OCTA can be used for clinical image interpretation.

DR affects millions of people globally, with projections suggesting this will grow more as diabetes prevalence continues to rise, disproportionately burdening low and middle-income regions where specialist ophthalmology access is scarce. Since OCTA devices remain expensive, from a clinical perspective, the proposed model is intended to function as a decision-support system rather than an autonomous diagnostic tool. For each examination, the model produces a probability score together with a binary prediction (DR or Normal). Patients classified as having a high probability of DR could be prioritized for comprehensive ophthalmic evaluation or referral, while clinicians would continue to interpret the original OCT images in conjunction with other clinical findings. Importantly, the multimodal fusion framework leverages complementary structural and vascular information to improve diagnostic confidence without requiring additional OCTA hardware when translated OCTA is available. By demonstrating that TR OCTA fusion achieves substantially higher sensitivity than OCT, this work shows that the hardware gap between well-resourced specialist clinics and underserved screening environments can be partly bridged through computation.

In summary, this work demonstrates that cross-modal attention fusion of OCT and OCTA enface images provides a substantial and consistent improvement over single-modal OCT classification for diabetic retinopathy detection. The TR OCTA variant, which eliminates the need for physical OCTA hardware achieves better performance across most of the experimental conditions including cross-dataset scenarios and matches or exceeds the performance reported by recent studies. Together, these findings validate cross-modal OCT–OCTA enface fusion as a clinically meaningful paradigm for automated DR screening and the TR OCTA pathway in particular as an alternative route toward accessible, high-sensitivity retinal disease detection.

## Data Availability

Publicly available datasets were analyzed in this study. This data can be found here: OCT: 10.1016/j.media.2024.103092 UIC: on request.
