# Supplementary material for: Cross-modal fusion of OCT and OCT angiography enface for improved diagnostics of diabetic retinopathy
Source: Front Med (Lausanne). 2026 Sep 29;13:1900570. doi: 10.3389/fmed.2026.1900570 (PMC13658096; doi:10.3389/fmed.2026.1900570)
Supplement: Supplementary file 1 [file Datasheet1.pdf]

Supplementary Material

1 SUPPLEMENTARY TABLE

Table S1. Early fusion classification performance (GT OCTA) with weighted loss

Training Arrangement	Accuracy	Precision	Recall	F1-Score	AUC	Specificity
OCT500 train UIC test	0.7040 (0.6990–0.7088)	0.9102 (0.9063–0.9137)	0.7211 (0.7160–0.7262)	0.8047 (0.8009–0.8084)	0.7377 (0.7321–0.7428)	0.6098 (0.5970–0.6233)
UIC train OCT500 test	0.8176 (0.8127–0.8225)	0.8004 (0.7737–0.8273)	0.1431 (0.1343–0.1530)	0.2428 (0.2293–0.2573)	0.7501 (0.7421–0.7584)	0.9908 (0.9895–0.9922)

Values in parentheses represent 95% bootstrap confidence intervals (1,000 iterations).

Table S2. Cross-modal fusion without attention classification performance (GT OCTA) with weighted loss

Training Arrangement	Accuracy	Precision	Recall	F1-Score	AUC	Specificity
OCT500 train UIC test	0.8872 (0.8838–0.8906)	0.9542 (0.9516–0.9567)	0.9100 (0.9067–0.9133)	0.9316 (0.9293–0.9338)	0.9450 (0.9428–0.9471)	0.7600 (0.7482–0.7715)
UIC train OCT500 test	0.8111 (0.8063–0.8159)	0.8370 (0.8110–0.8610)	0.1000 (0.0920–0.1087)	0.1787 (0.1658–0.1920)	0.8100 (0.8028–0.8170)	0.9950 (0.9939–0.9960)

Values in parentheses represent 95% bootstrap confidence intervals (1,000 iterations).

Table S3. Cross-modal fusion classification performance (GT OCTA) after domain adaptation with weighted loss

Training Arrangement	Accuracy	Precision	Recall	F1-Score	AUC	Specificity
OCT500 train UIC test	0.8500 (0.8463–0.8538)	0.8716 (0.8677–0.8750)	0.9649 (0.9628–0.9671)	0.9159 (0.9135–0.9180)	0.8231 (0.8173–0.8286)	0.2201 (0.2096–0.2313)

Values in parentheses represent 95% bootstrap confidence intervals (1,000 iterations).

2 SUPPLEMENTARY INFORMATION

TR OCTA was generated using a Brownian Bridge Diffusion Model (BBDM) implemented in the latent space of a pretrained VQGAN (Figure S1). The diffusion network used a 2D U-Net backbone with six input channels and three output channels. The model directly learns the stochastic transition between OCT and OCTA latent representations through Brownian Bridge conditioning. Training used 1,000 diffusion

steps, while accelerated inference was performed using a linear bridge sampling schedule with 20 sampling steps.

The translation model was trained exclusively on paired OCT/OCTA data from the OCT500 dataset, with the 3-mm and 6-mm subsets processed separately. A 70/5/25% training/validation/test split was used. The independent UIC dataset was not used for training rather was used to evaluate cross-dataset generalization. Images were processed at a spatial resolution of 224 × 224 pixels.

The BBDM was trained for 100 epochs using the Adam optimizer with an initial learning rate of 1×10^{-4} and $\beta_1 = 0.9$. A learning-rate scheduler with a decay factor of 0.5, patience of 10 epochs and minimum learning rate of 5×10^{-7} was used. Gradient accumulation was set to two batches, and exponential moving averaging of model weights was applied with a decay of 0.995.

Translation quality was evaluated by comparing TR OCTA with GT OCTA using structural similarity index (SSIM), Fréchet inception distance (FID) and perceptual contrast quality index (PCQI). In addition, vascular fidelity was assessed using blood vessel density, blood vessel caliber, blood vessel tortuosity and vessel perimeter index.

2.1 Figures

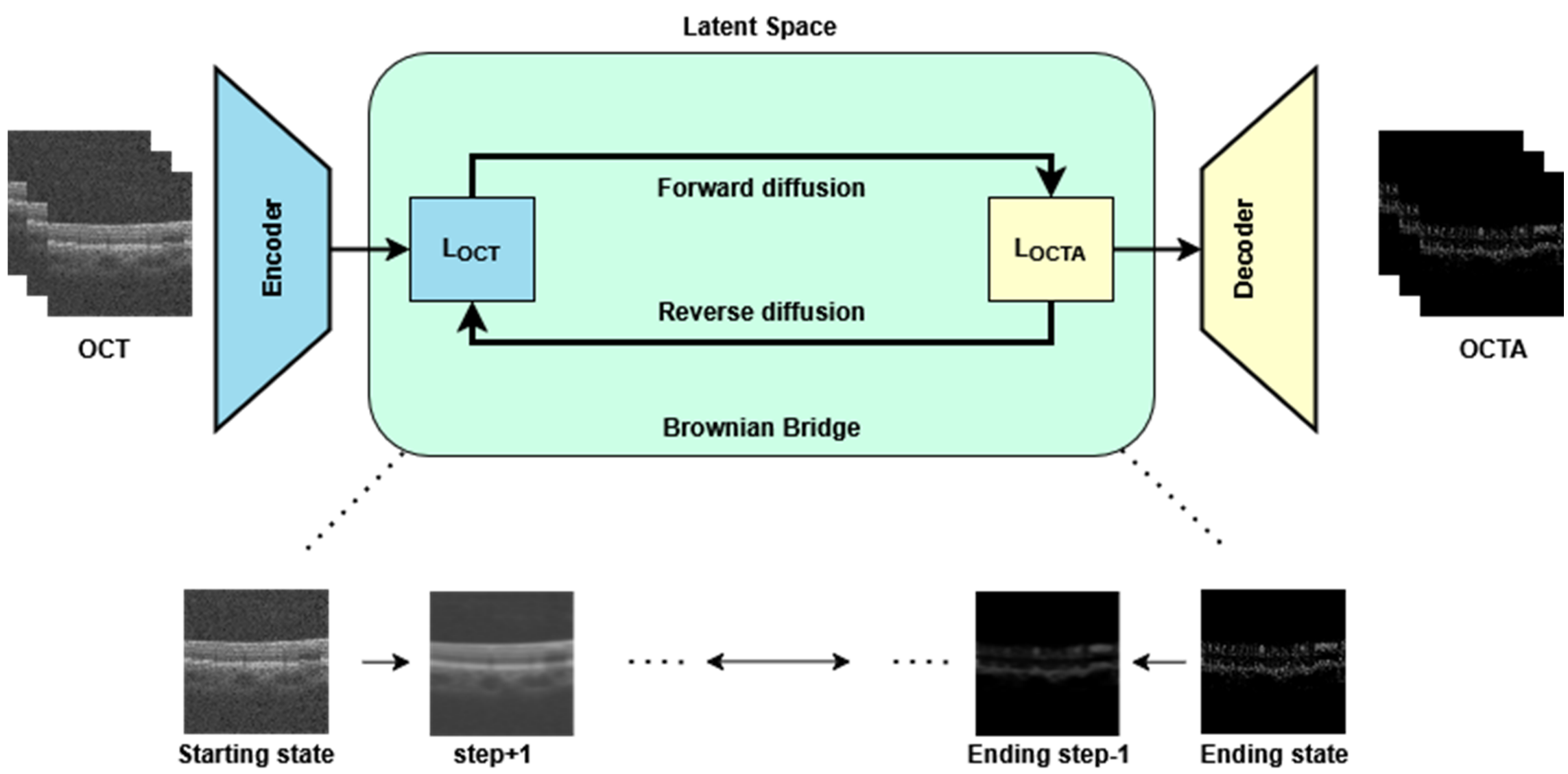

Figure S1. Diffusion model translation framework

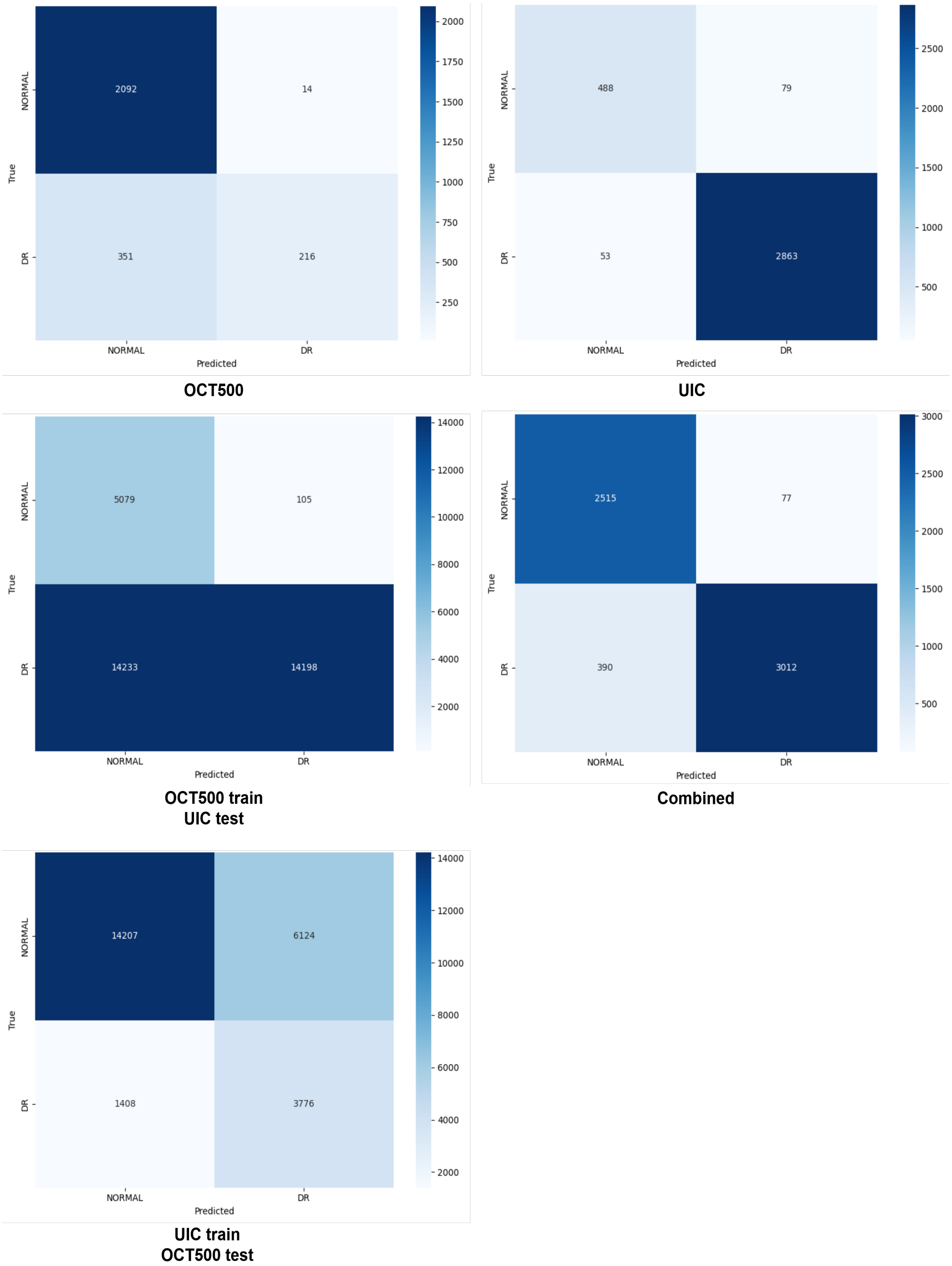

Figure S2. Confusion matrices for the base model.

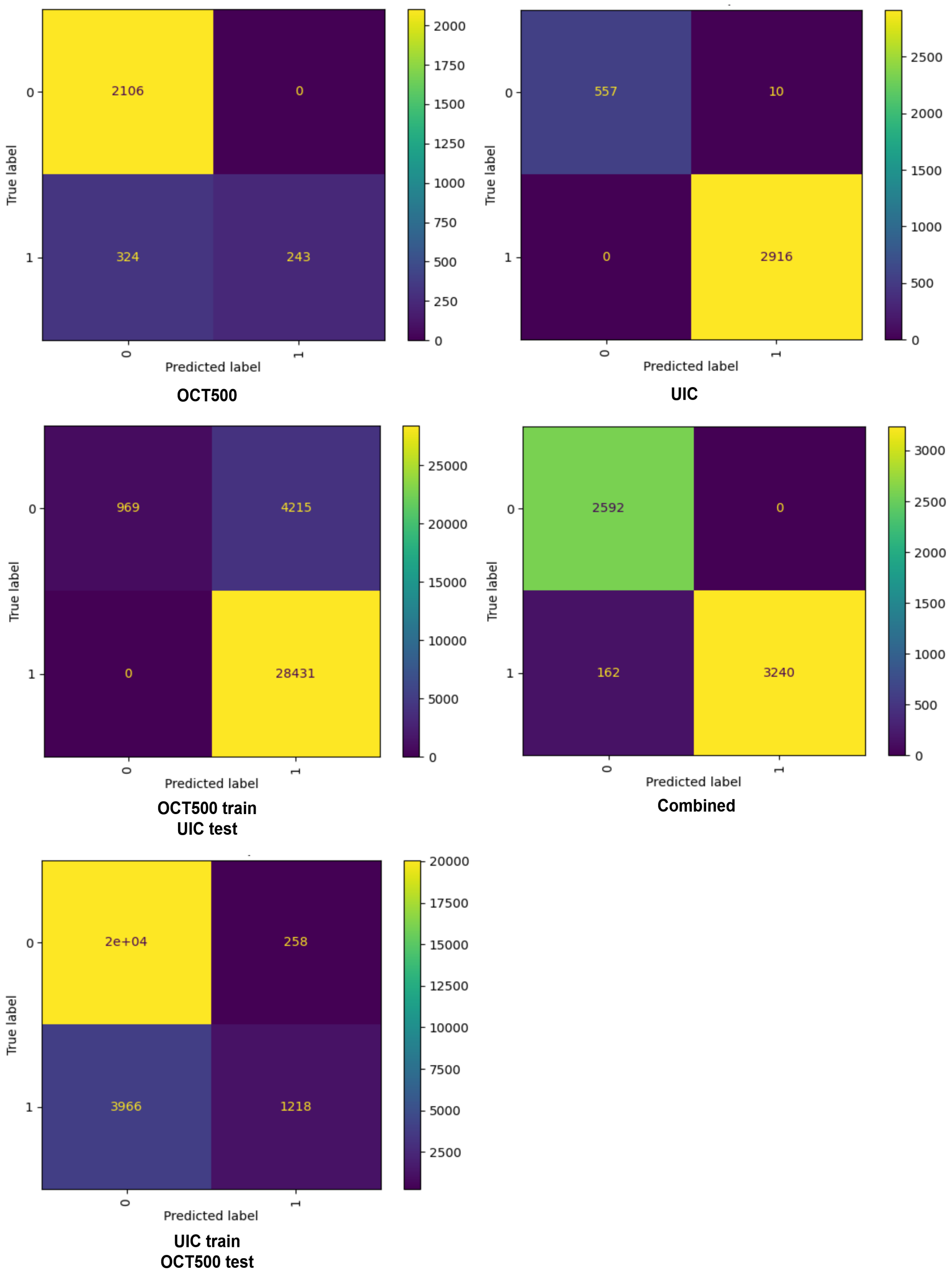

Figure S3. Confusion matrices for OCT-OCTA (GT) enface fusion model.

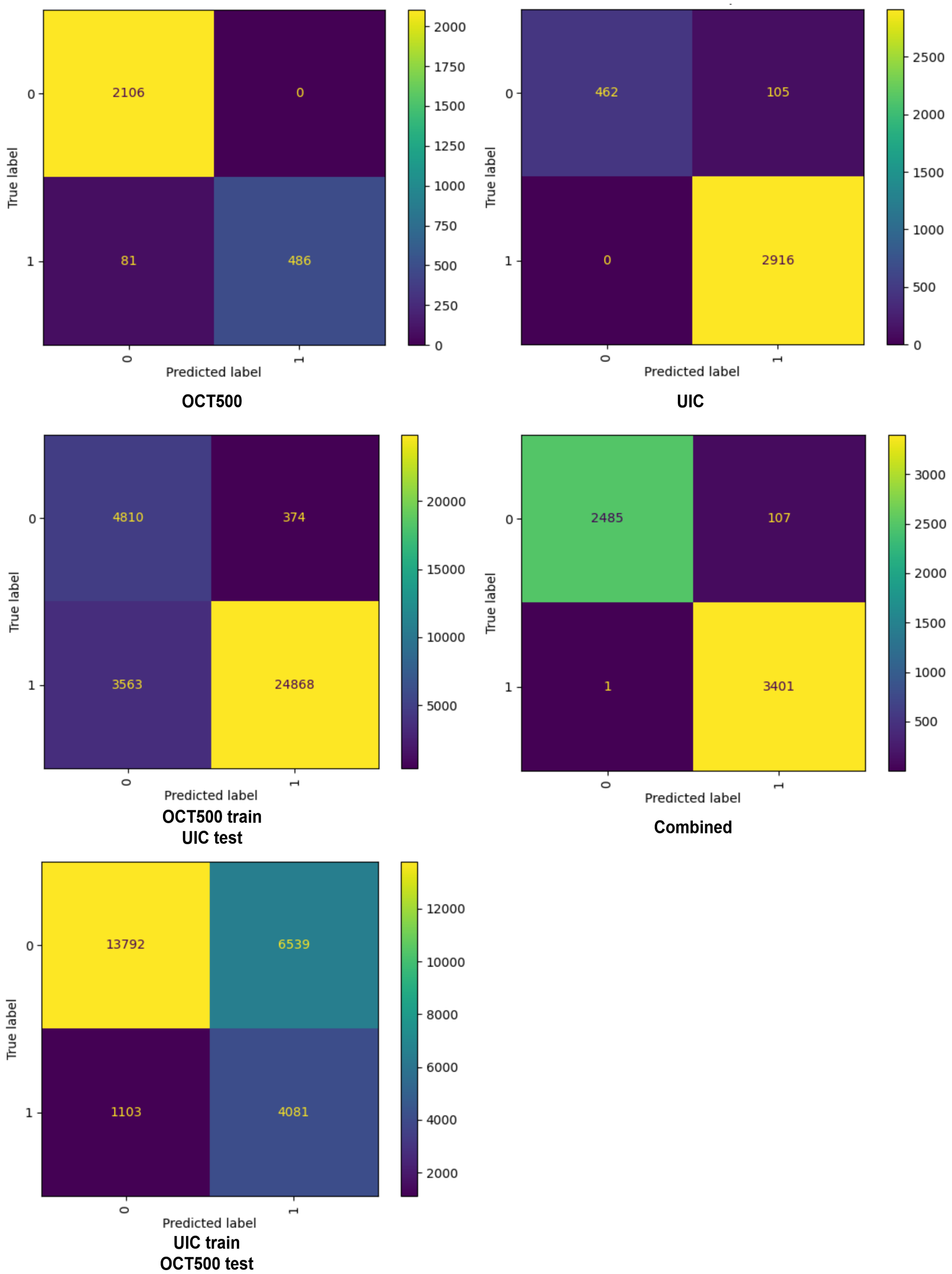

Figure S4. Confusion matrices for OCT-OCTA (TR) enface fusion model.

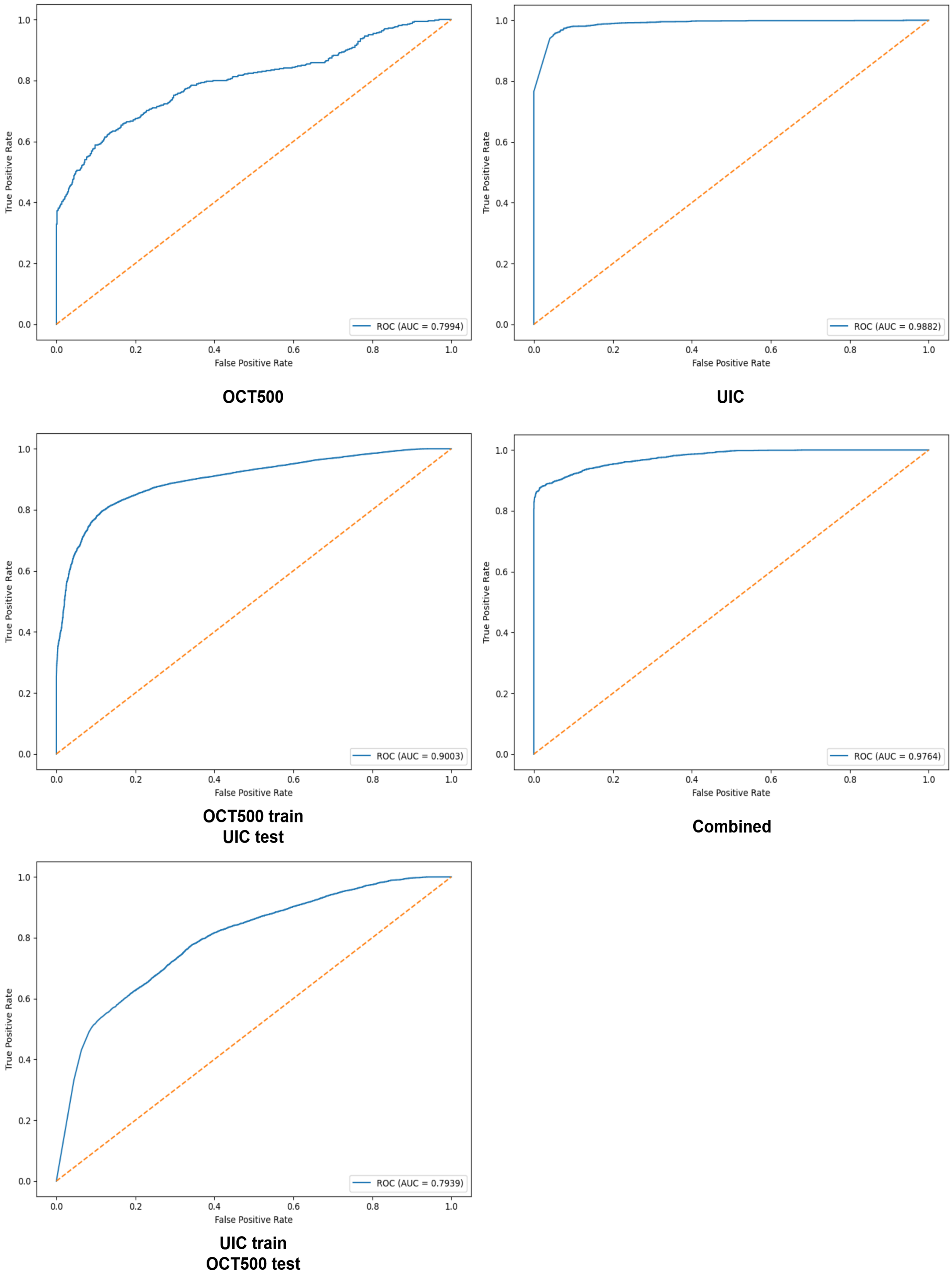

Figure S5. ROC curves for the base model.

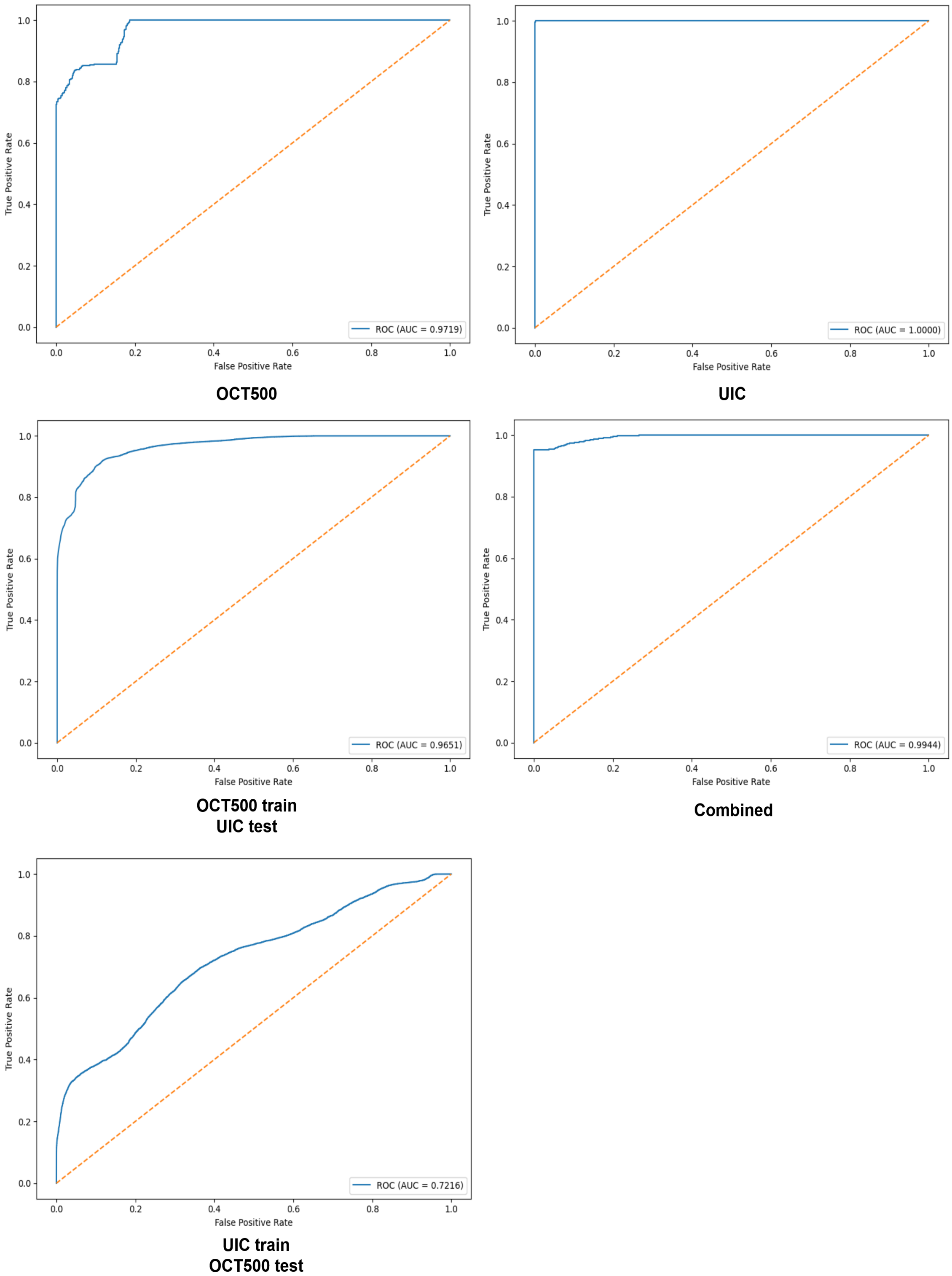

Figure S6. ROC curves for OCT-OCTA (GT) enface fusion model.

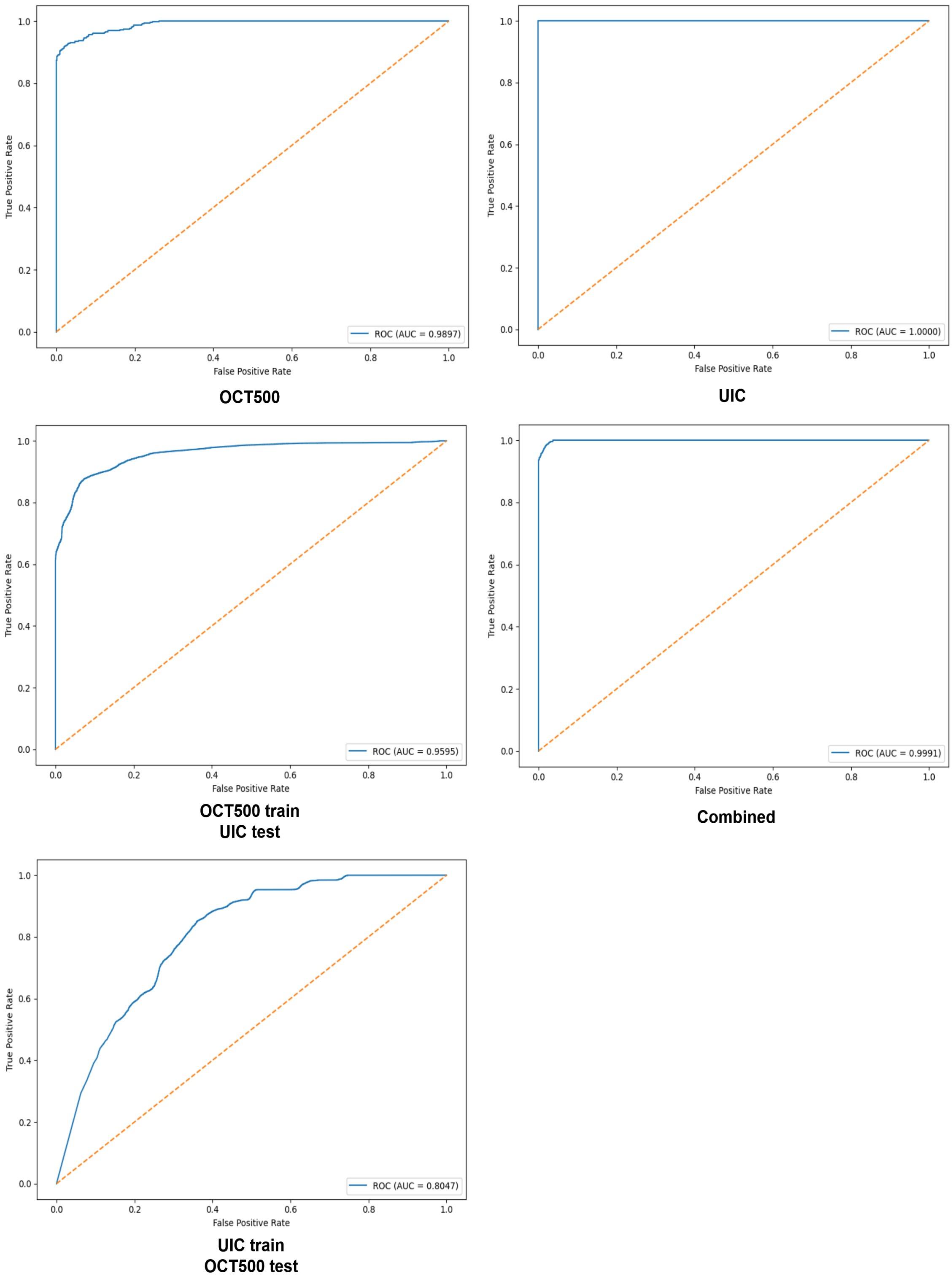

Figure S7. ROC curves for OCT-OCTA (TR) enface fusion model.
